# Exploring Peoples' Perception of Autonomy and Reactance in Everyday AI Interactions

**DOI:** 10.3389/fpsyg.2021.713074

**Published:** 2021-09-29

**Authors:** Supraja Sankaran, Chao Zhang, Henk Aarts, Panos Markopoulos

**Affiliations:** ^1^Department of Industrial Design, Eindhoven University of Technology, Eindhoven, Netherlands; ^2^Department of Psychology, Utrecht University, Utrecht, Netherlands

**Keywords:** autonomy, reactance, artificial intelligence, explainability, human-centered AI

## Abstract

Applications using Artificial Intelligence (AI) have become commonplace and embedded in our daily lives. Much of our communication has transitioned from human–human interaction to human–technology or technology-mediated interaction. As technology is handed over control and streamlines choices and decision-making in different contexts, people are increasingly concerned about a potential threat to their autonomy. In this paper, we explore autonomy perception when interacting with AI-based applications in everyday contexts using a design fiction-based survey with 328 participants. We probed if providing users with explanations on “why” an application made certain choices or decisions influenced their perception of autonomy or reactance regarding the interaction with the applications. We also looked at changes in perception when users are aware of AI's presence in an application. In the social media context, we found that people perceived a greater reactance and lower sense of autonomy perhaps owing to the personal and identity-sensitive nature of the application context. Providing explanations on “why” in the navigation context, contributed to enhancing their autonomy perception, and reducing reactance since it influenced the users' subsequent actions based on the recommendation. We discuss our findings and the implications it has for the future development of everyday AI applications that respect human autonomy.

## Introduction

The digital era has brought in a massive transformation to the way we communicate and interact with people and technologies. The rapid development of artificial intelligence (AI) and its integration in everyday applications has been key to this transformation. Artificial intelligence includes a diverse spectrum of paradigms covering algorithms using fundamental logic-based probabilistic methods, complex deep learning methods, and distributed AI using multi-agent autonomous systems (Finlay and Dix, 1996; Nilsson, 2014). In this paper, we refer to AI-based applications as applications that employ some degree of machine learning to understand people's behavior and present information. Diverse AI-based applications are now embedded in our daily lives from domotics such as smart thermostats and robotic vacuum cleaners that simplify chores, entertainment, and shopping platforms that guide our choices, navigation systems to maneuver through traffic optimally, social media apps that have transformed communication methods, and typing assistants that are enabling us to write emails quickly or fill out our resumes automatically. As these applications are gradually supplanting human-decision making, streamlining choices, and modifying communication methods, people gradually begin to perceive a threat to their autonomy (André et al., 2018; Sankaran et al., 2020a,b; Pizzi et al., 2021).

### Understanding Autonomy and Reactance

Autonomy forms a fundamental aspect of human well-being and development and is defined as an individual's capacity for self-determination and governance (Dryden, 1995). According to self-determination theory, a person is considered autonomous if their choices, decisions, and actions are instigated and endorsed by their conscious self (Deci and Ryan, 1985; Ryan and Deci, 2000). Likewise, it is also characterized by a person independently making an informed decision after carefully evaluating all choices and options (Hodgins and Knee, 2002). Therefore, if technology constrains choices, takes over control of decisions and actions, or modifies normal communication methods, people's perception of autonomy could be seriously jeopardized. This has been cited as one of the critical challenges of AI development in the future (Anderson and Rainie, 2018). The need to take a broader look at autonomy has been identified as crucial for trust repair in human–machine interactions (de Visser et al., 2018). The need to respect human autonomy to develop trustworthy AI has also been emphasized in regulatory guidelines by the European Union (AI HLEG, 2019; European Commission, 2021).

The negation of autonomy causes reactance. According to reactance theory, reactance is a motivational state that is caused by a certain threat to freedom and invokes the need to reassert free behavior (Brehm, 1966, 1989). Recent research states reactance as negative motivational arousal which is experienced by people when they experience a threat to or loss of free behavior and independent actions (Steindl et al., 2015). In human–computer interaction, factors such as reduced freedom of choice and behavior restriction cause psychological reactance when interacting with systems in real-life situations (Ehrenbrink and Prezenski, 2017). Furthermore, studies have shown that digital assistants influence users' perceived control during an interaction, often resulting in disappointment with the online recommendation (André et al., 2018) and experience a psychological reactance when they perceive that their freedom is reduced (Lee and Lee, 2009). Multiple studies have also explored how AI impacts trust in machines and have provided approaches to repair trust in human–machine interactions (de Visser et al., 2018; Kulms and Kopp, 2019; Hayes and Moniz, 2021). Yet, there is a lack of a comprehensive look at autonomy perception or reactance encompassing various factors such as trust, control, freedom of choice, and decision-making support. Therefore, we sought for a widely encompassing consideration of everyday contexts to understand how users perceive potential threats to their autonomy and whether they experience psychological reactance while interacting with AI systems.

### Motivation

The fundamental objective of our exploratory study was to gain a comprehensive understanding of people's perceptions of autonomy and reactance when interacting with AI in different everyday contexts. When interacting with intelligent agents, researchers have been looking into how people's perception of communication varies as compared to interaction with other humans or human-like agents. People perceived a higher reactance when interacting with non-human like digital assistance especially when the interaction is initiated by the AI agent (Pizzi et al., 2021). In a series of studies where participants saw predictions made by an algorithmic forecaster, a human forecaster, the combination thereof, or were not told who made the predictions, they preferred a human forecaster and perceived the algorithmic forecaster to be *inferior* even if the algorithmic forecaster outperformed the human forecaster (Dietvorst et al., 2015). In a study assessing social perceptions in human–AI collaboration, participants found that collaborating with human partners was more *likable*, facilitated better rapport and was perceived as a *more intelligent* partner as compared to an AI partner (Ashktorab et al., 2020).

Similarly, people experienced algorithmic anxiety owing to a lower degree of trust, confidence, and acceptance of recommendations made by AI agents since they did not fully understand those (Jhaver et al., 2018; Kulms and Kopp, 2019). To overcome issues of algorithmic aversion and anxiety, researchers often look upon explainable AI as a means to increase trust and confidence in recommendations made by AI agents (Ribeiro et al., 2016; Alexandrov, 2017). From a societal perspective, organizations such as the European Union have also developed regulations on algorithmic decision-making and the right to an explanation (Goodman and Flaxman, 2017).

In the context of everyday interactions, factors such as intelligibility, control over a system, trust, likeability, or acceptance and expectations of the system are also shown to influence people's perception of autonomy or reactance (Sankaran and Markopoulos, 2021). An extensive literature review and survey in human–computer interaction showed that factors like undesirable behavior of agents, restricting choice, and control would lead to psychological reactance among users (Ehrenbrink and Prezenski, 2017). However, it is not clear how these factors influence people's perception of autonomy and reactance vary in different contexts. Therefore, alongside understanding people's perceptions of autonomy in different everyday contexts, we hypothesized that their perceptions might vary in two distinct factors: (F1)—when they received explanations on why a specific recommendation was made, and (F2)—when their attention was specifically drawn to the presence of AI. In the present study, we manipulate explanations by using brief personalized textual explanations which are known to be effective in recommender systems (Tintarev and Masthoff, 2012). To effectively manipulate people's attention to the presence of AI we use a textual cue alongside a symbolic visual stimulus as adopted in prior research (Jakesch et al., 2019).

This paper presents the outcomes from our exploratory survey and highlights directions for future development of AI applications to respect human autonomy.

## Methods

### Design

Design fiction is a method aimed at exploring possible future interactions by creating speculative scenarios using design artifacts (Grand and Wiedmer, 2010; Dunne and Raby, 2013). It enables people to get a more realistic understanding of a hypothesized idea and thereby enable a more critical reflection using artifacts that blur the boundary between fact and fiction (Bleecker, 2009, 2010). Theater and film are commonly used media in design fiction research to transport participants to the hypothesized speculative future to enable them to surface critical questions, overcome pre-existing assumptions and notions about technology, and expand their sense of what is possible (Briggs et al., 2012; Auger, 2013; Dunne and Raby, 2013). This method has been successfully applied in many research studies specifically in the field of human-computer interaction (Wakkary et al., 2013; Blythe, 2014). Recently, researchers are also adopting design fiction as an approach for studying the ethical implications of technological innovations (York and Conley, 2020).

Therefore, to test our hypotheses, we adopted a video-based design fiction approach and carried out a 2 × 2 between-subjects design to study the distinction in the two factors (F1 and F2) (Table 1).

**Table 1:** Overview of manipulations in the study design.

	**Attention drawn**	**Attention not drawn**
Explained	**Group A–E** •The attention of participants in this group was intentionally drawn to the presence of AI using visual and textual cues. •They **received explanations** on *why* specific suggestions/options are provided.	**Group NA–E** •The attention of participants in this group was not drawn to the presence of AI. •They **received explanations** on *why* specific suggestions/options are provided.
Not explained	**Group A–NE** •The attention of participants in this group was intentionally drawn to the presence of AI using visual and textual cues. •They **did not receive explanations** on *why* specific suggestions/options are provided.	**Group NA–NE** •The attention of participants in this group was not drawn to the presence of AI. •They **did not receive explanations** on *why* specific suggestions/options are provided.

We selected eight diverse everyday application scenarios based on applications that were commonly used by most people (Pega, 2018). Within each group, participants were presented with all eight application contexts in random order—(A1) a movie recommender system, (A2) a hotel booking assistant, (A3) a climate-control thermostat, (A4) a navigation system, (A5) a social media app, (A6) an email composer with an auto-correct feature, (A7) a fitness app, and (A8) an online shopping website.

Apart from participants rating their agreement to statements on perceived autonomy and reactance in each scenario, we also assessed the effectiveness of the manipulations (i.e., did the perceive the presence of AI and the “why” explanations). Additionally, we also presented participants with open-ended questions to probe if and why they felt a certain application was relatively more or less intelligent to get a nuanced understanding of their perceptions.

### Sample Size Calculation

The sample size was determined by a power analysis using the R package *Superpower* (Caldwell et al., 2021). We specified the groups means of the four conditions based on two expectations: (1) awareness of the presence of AI and providing explanation should both increase perceived autonomy (and reduce reactance) but there should be no interaction effect; (2) the main effect of providing explanation would be larger than the main effect of making them aware of the presence of AI. We also assumed a standard deviation of 1 for all conditions. These specifications led to two main effects of the sizes of Cohen's *f* = 0.15 (awareness) and 0.30 (explanation). Power analysis showed that to have approximately 80% power at the alpha level of 0.05 for the smaller effect (Cohen's *f* = 0.15), 80 participants were needed for each condition. The code for the power analysis can be found on the project repository at Open Science Framework (section Data Availability Statement).

### Participants

Participants were recruited using the Prolific^1^ crowdsourcing platform. We aimed for a large diversity of participants who have sufficient experience in interacting with AI based applications in their everyday lived. The majority of the participants were male, in the age category of 18–24 years, and had at least a high school degree or equivalent (Table 2). Each participant received a compensation of 4.39 British pounds consistent with Prolific norms.

**Table 2:** Overview of participant demographics.

	***N***	**%**
**Total participants**	328	
- Group made aware and explained (A-E)	82	25
- Group made aware but not explained (A-NE)	82	25
- Group not made aware but explained (NA-E)	82	25
- Group not made aware and not explained (NA-NE)	82	25
**Gender**		
- Male	218	66.5
- Female	107	32.6
- Non-Binary	3	0.9
**Age**		
−18–24	190	57.9
−25–34	98	29.9
−35–44	22	6.7
−45–54	12	3.7
−55 and above	6	1.8
**Education**		
- Less than high school	9	2.7
- High school degree or equivalent	157	47.9
- Bachelor's degree	94	28.7
- Master's degree	55	16.8
- Doctorate	7	2.1
- Other	6	1.8

### Experimental Task Using Design Fiction

**Figure 1:**
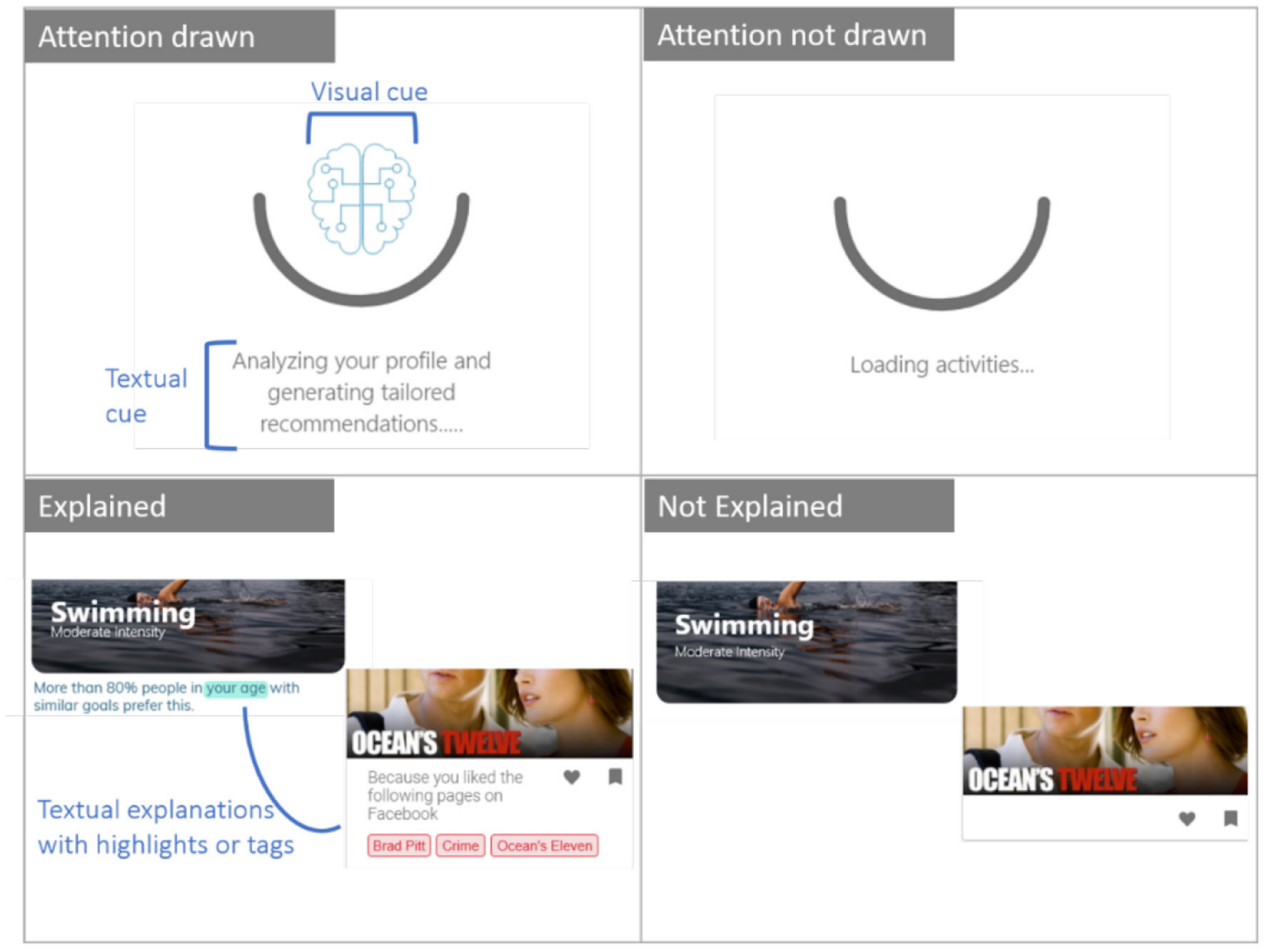
Overview of designs showing the manipulations that were used for the experiment.

For each of the eight application scenarios, we designed fictitious interfaces which mimicked related real-life applications (section Data Availability Statement). For example, the movie recommender system resembled Netflix's interface, the social media feed resembled the Twitter interface, the email composer resembled Gmail's interface, etc. The manipulations were embedded in the interface design of the different applications (Figure 1).

Participants viewed different versions of the fictitious interfaces based on the manipulation group they were assigned to. These are design fictions because they showed features that were speculations and depicted potential future designs which are not present in current interfaces unlike in scenario-based studies. Participants who were primed about the presence of intelligence saw a visual cue (an electronic brain-like icon indicating AI) as well a text indicating that the application was analyzing their data or information before making a recommendation or prediction. Visual priming as a technique is shown to influence users' perceptions in human–agent interaction (Sanoubari et al., 2018). Explanations were designed in the interface as textual information with highlights or pointers as to “why” a certain content was being shown on the application interface. Since the experiment was conducted in the form of a survey, participants could not directly interact with the different fictitious interfaces. Therefore, they were shown video snippets of a user interacting with the different interfaces starting from opening the application to viewing the recommendations or outcomes. At the end of watching a video snippet of a user interacting with each application, participants were presented with a series of 10 questions and asked to rate how they perceived autonomy and reactance as they observed from the user interaction with that application.

### Measurements

Both perceived autonomy and reactance were measured by five items each. Participants rated 10 statements corresponding to them using five-point Likert scales (1 = Strongly disagree, 5 = Strongly agree) (Table 3).

**Table 3:** Statements to assess perceived autonomy and reactance.

****Perceived autonomy** (average Cronbach's alpha = 0.83 across applications)**	****Perceived reactance** (average Cronbach's alpha = 0.78 across applications)**
1. The system provided choices based on the user's true interests.	6. The system (which interacts as illustrated) might frustrate the user because it does not let them make free and independent decisions.
2. The system let the users do things their own way.	7. The system might irritate the user when the system points out things that are obvious to them.
3. The system helped the user take actions that they wanted to do rather than because they were told to.	8. Users might become angry when the system restricted their freedom of choice.
4. The system let the user be in control of what they did.	9. Users might consider recommendations from the system (such as a filtered list of options, alternatives etc.) as an intrusion.
5. The system helped the user make their own decisions.	10. Advice and recommendations might induce the user to do just the opposite.

These statements were adapted from measures of general autonomy as used by Sheldon et al. (2001) and the 11-point Hong reactance scale (Hong and Faedda, 2016). Based on the satisfactory to good internal reliabilities, single scores of perceived autonomy and reactance were computed by aggregating the scores on their corresponding five items. While perceived autonomy and reactance certainly correlated negatively (i.e., a person perceives less autonomy while showing stronger reactance to recommendation), these correlations were only moderate across the eight application domains (Person's *r* in the range of −0.4 and −0.53). This justified the distinctiveness of the two constructs and their separate treatments in the data analyses.

Apart from statements collecting participants' perceived autonomy and reactance in each scenario, we also included two five-point Likert scale-based manipulation checks for the explainability (i.e., “In the videos, the applications gave clear explanations to the user on why certain options/recommendations were provided.”) and awareness (i.e., “In the videos, the system shown used AI to generate options/recommendations.”). Additionally, we also presented participants with open-ended questions to probe if and why they felt a certain application was relatively more or less intelligent to get a nuanced understanding of their perceptions.

### Data Analysis

For each of the dependent variables (i.e., perceived autonomy and psychological reactance), we first estimated the overall effects of awareness of presence of AI (awareness) and explanation across all the scenarios. For the overall effects, a linear mixed model was fitted to predict perceived autonomy or reactance, including awareness, explanation, and their interaction term as predictors, as well as random intercepts for the participants. To facilitate interpretation, we used deviation coding for the predictors so that the effects would indicate the increase or decrease in the outcome variable (perceived autonomy or reactance) when awareness was used or explanation was provided, compared with the average level of the outcome variable (e.g., an effect of 0.5 actually meant a difference of 1 point between the explanation and no-explanation condition). To examine whether the role of application scenario, in a second model we added application scenario as an additional predictor in the previous linear mixed model. This new model could answer the questions whether certain scenarios would lead to higher autonomy or less reactance regardless of the manipulations (its main effect) and whether the effects of awareness and explanation were application-dependent (interaction effects). Marginal *R*^2^ was used to evaluate whether adding application scenario as a factor substantially increased the explanatory power of the models.

In case the application scenario would not be found to play an important role, we would continue with analyses for the eight specific scenarios. A simple regression model was fitted for each scenario, with either perceived autonomy or reactance as the outcome variable, and awareness, explanation, and their interaction term as the predictors. Because each of the effects of interest was tested eight times, we applied Bonferroni-corrected alpha level of 0.05/8 = 0.00625 to identify significant effects. All the statistical analyses were performed using the R statistical software (version 4.03; R Core Team, 2020).

## Results

### Manipulation Check

Welch two-sample *t*-tests were used to perform the manipulation checks. Participants in the awareness conditions indeed judged the systems they interacted with as having AI to a greater extent (*M*_*awareness*_ = 3.90, *SD*_*awareness*_ = 0.35) than participants in the no-awareness condition (*M*_*no*−*awareness*_ = 3.76, *SD*_*no*−*awareness*_ = 0.54), mean difference = 0.14, 95% CI = [0.04, 0.24], *p* = 0.006. Similarly, participants in the explanation conditions judged the systems they interacted with as providing explanations to a greater extent (*M*_*explanation*_ = 3.67, *SD*_*explanation*_ = 0.35) than their peers in the no explanation condition (*M*_*explanation*_ = 2.79, *SD*_*no*−*explanation*_ = 0.35), mean difference = 0.89, 95% CI = [0.83, 0.95], *p* < 0.001. In contrast, the manipulation of awareness did not influence the perceived extent of explanation and the manipulation of explanation did not influence the perceived extent of intelligence (both *p*s > 0.27). These results confirmed the effectiveness and distinctiveness of the manipulations, even though the manipulation of awareness seemed to be rather weak.

### Overall Effects of Explanation and Awareness of AI on Perceived Autonomy

**Figure 2:**
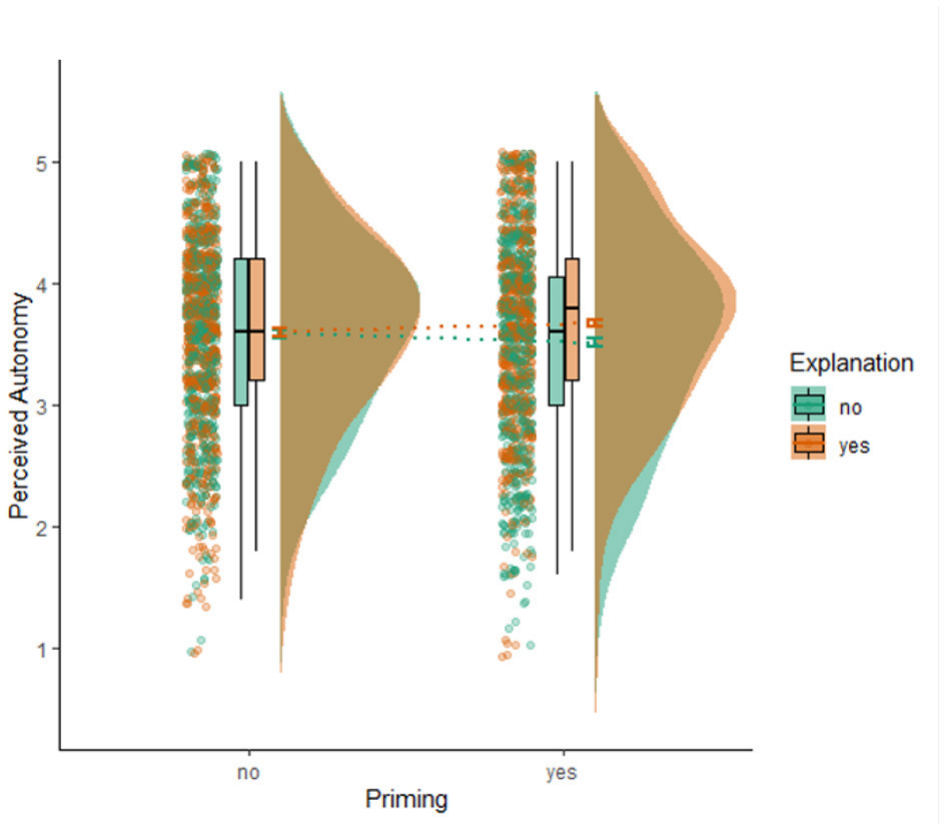
Visualization of means, standard errors, boxplots, distributions, and raw data points of perceived autonomy for the four experimental conditions.

Table 4 shows the group means and standard deviations for perceived autonomy (also see Figure 2 for the visualized group means and raw data points). For perceived autonomy, linear mixed modeling indicated no main effect of awareness (*B* = −0.0003, 95% CI = [−0.05, 0.05], *p* = 0.99), no main effect of explanation (*B* = 0.04, 95% CI = [−0.01, 0.09], *p* = 0.092), and no interaction effect (*B* = 0.04, 95% CI = [−0.01, 0.08], *p* = 0.152).

**Table 4:** Means and standard deviations (in brackets) of perceived autonomy for each of the experimental conditions.

	**No-awareness**	**Awareness**
No-explanation	3.59 (0.77)	3.51 (0.86)
Explanation	3.60 (0.80)	3.67 (0.78)

Adding application scenario term into the models increased the percentage of variance explained in the dependent variables substantially (marginal *R*^2^ = 0.078). Since the application scenario variable had eight different levels (i.e., eight different AI applications), the full modeling results included as many as 31 fixed effects. For brevity, here we report the effects that were larger than 0.1 (on five-point scales) but interested readers could replicate the full results by using the shared data and analysis script on Open Science Framework. First, it seemed that participants perceived different levels of autonomy in the different application scenarios, regardless of whether awareness of AI was used or whether explanation had been provided. Compared to the average level of perceived autonomy, perceived autonomy was significantly higher in the scenarios of car navigation (*B* = 0.22, 95% CI = [0.15, 0.29], *p* < 0.001) and fitness recommendation (*B* = 0.29, 95% CI = [0.22, 0.35], *p* < 0.001) but was significantly lower in the scenario of social media consumption (*B* = −0.27, 95% CI = [−0.34, −0.20], *p* < 0.001) and house climate control (*B* = −0.11, 95% CI = [−0.18, −0.04], *p* = 0.001). In addition, some of the effects of awareness and explanation on the perceived autonomy were moderated by the application scenario. For the application of travel booking assistance, the awareness of intelligence and data processing had a slightly larger role in increasing perceived autonomy (*B* = 0.10, 95% CI = [0.04, 0.17], *p* = 0.003), compared with the average effects of awareness across all applications. Compared to the average effect of explanation on perceived autonomy across all applications, providing an explanation had a significantly larger positive influence on autonomy for the car navigation system (*B* = 0.22, 95% CI = [0.15, 0.28], *p* < 0.001), but a less positive influence for the house climate control system (*B* = −0.14, 95% CI = [−0.21, −0.08], *p* < 0.001) and the fitness recommendation system (*B* = −0.11, 95% CI = [−0.18, −0.04], *p* = 0*.0*01). Finally, there were no statistically significant three-way interaction effects on perceived autonomy with any of the application scenarios.

### Overall Effects of Explanation and Awareness of AI on Psychological Reactance

**Figure 3:**
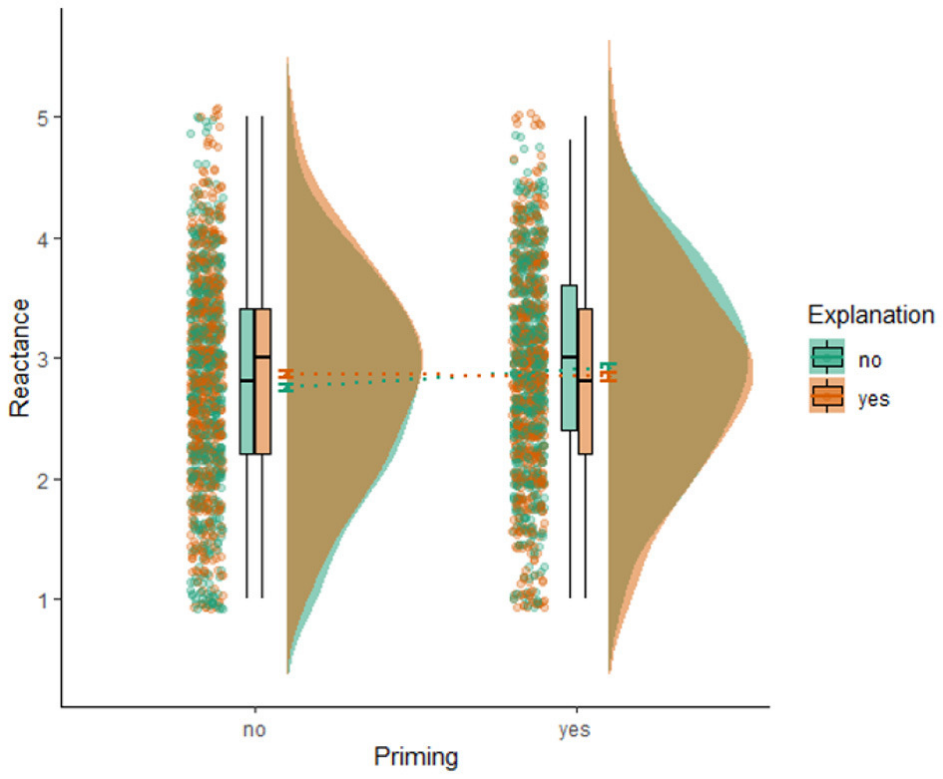
Visualization of means, standard errors, boxplots, distributions, and raw data points of reactance for the four experimental conditions.

Table 5 shows the group means and the standard deviations for perceived reactance (also see Figure 3 for the visualized group means and raw data points). For reactance, results also indicated no main effect of awareness (*B* = 0.04, 95% CI = [−0.02, 0.09], *p* = 0.21), no main effect of explanation (*B* = 0.01, 95% CI = [−0.05, 0.07], *p* = 0.72), and no interaction effect (*B* = −0.05, 95% CI = [−0.10, 0.01], *p* = 0.12). For both dependent variables, the fixed effects explained little variance (both marginal *R*^2^ = 0.005).

**Table 5:** Means and standard deviations (in brackets) of psychological reactance for each of the experimental conditions.

	**No-awareness**	**Awareness**
No-explanation	2.75 (0.85)	2.91 (0.81)
Explanation	2.86 (0.86)	2.84 (0.85)

Adding application scenario into the models increased the percentage variance explained in the dependent variables to a substantial extent (marginal *R*^2^ = 0.074). Compared to the average level of reactance, participants showed significantly higher reactance when receiving social media filters (*B* = 0.27, 95% CI = [0.20, 0.33], *p* < 0.001), but significantly lower reactance when receiving navigation advices (*B* = −0.26, 95% CI = [−0.33, −0.20], *p* < 0.001), fitness recommendations (*B* = −0.27, 95% CI = [−0.34, −0.20], *p* < 0.001), and automatic house climate adjustments (*B* = −0.13, 95% CI = [−0.20, −0.06], *p* < 0.001). Moreover, for the application of the travel booking assist, priming the awareness of intelligence and data processing had a slightly larger role in decreasing reactance (*B* = −0.10, 95% CI = [−0.17, −0.03], *p* = 0.003). Compared to the average effect of explanations on reactance across all applications, providing explanation reduced reactance even more for car navigation system (*B* = 0.19, 95% CI = [0.12, 0.26], *p* < 0.001), but less for house climate control system (*B* = 0.12, 95% CI = [0.05, 0.18], *p* < 0.001). Finally, there were no statistically significant three-way interaction effects on reactance with any of the application scenarios.

### Specific Effects on the Perceived Autonomy and Reactance for each of the Application Scenarios

**Figure 4:**
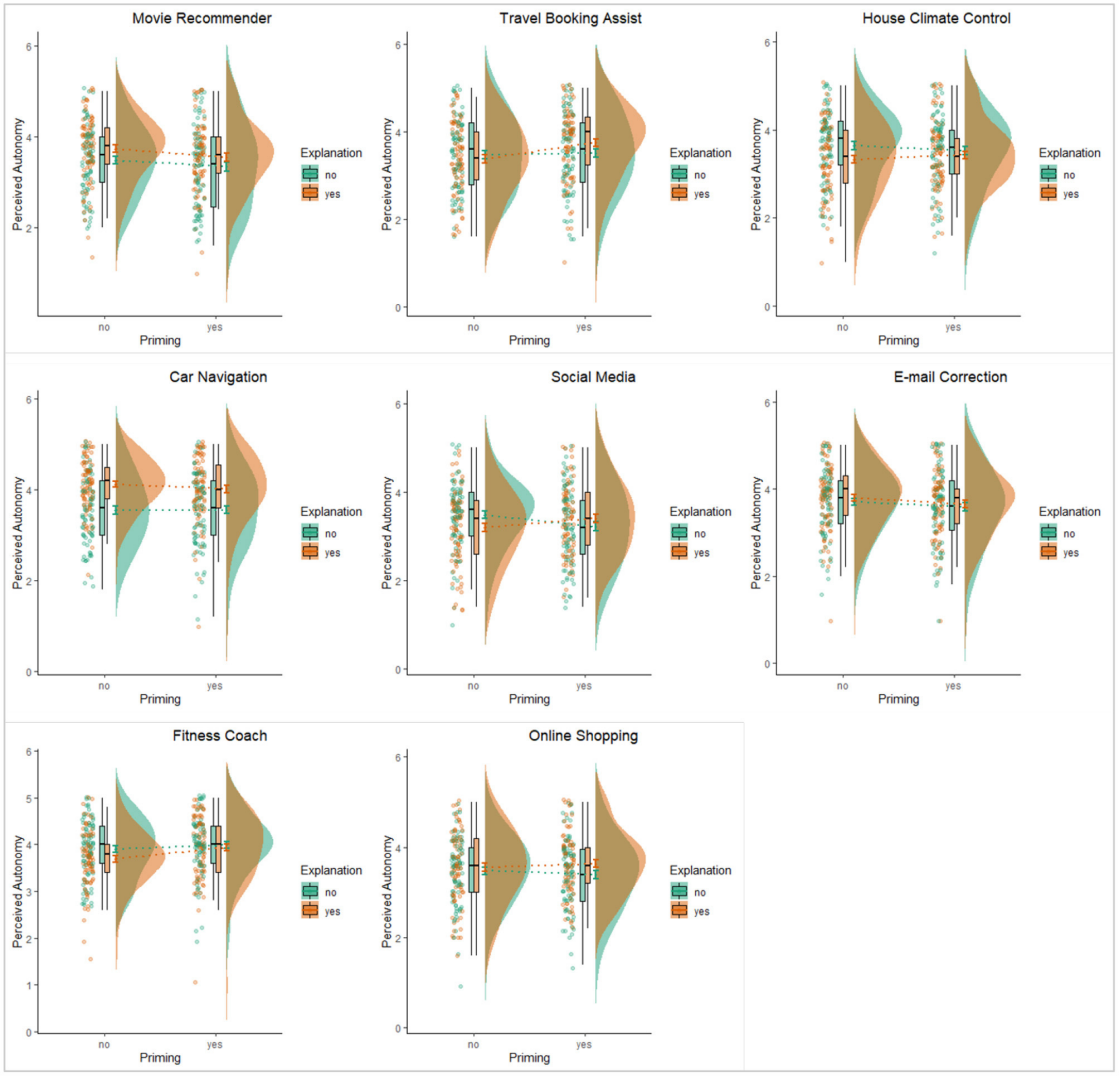
Visualization of the effects of explanation and awareness on perceived autonomy for the eight specific application scenarios.

**Figure 5:**
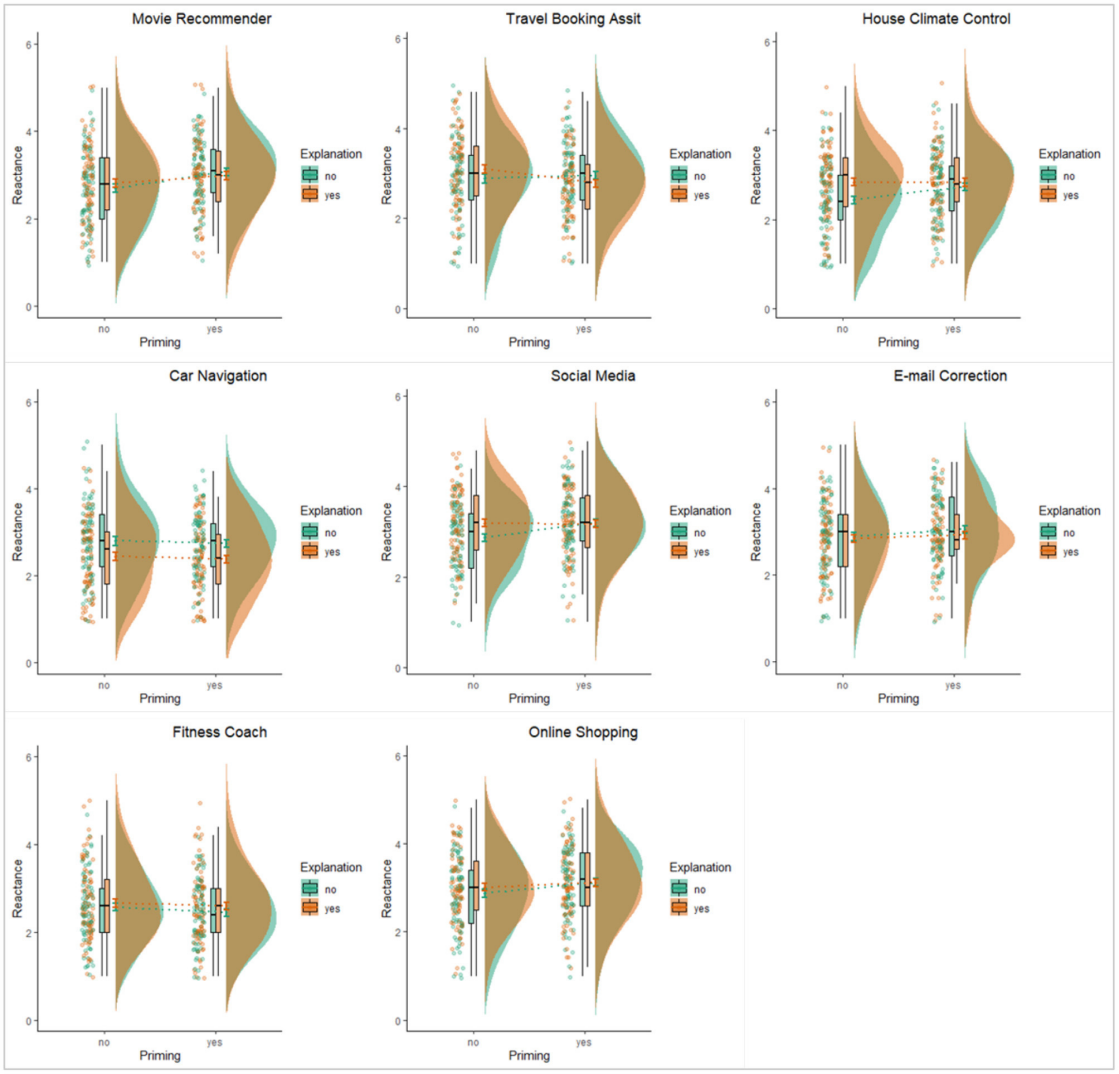
Visualization of the effects of explanation and awareness on reactance for the eight specific application scenarios.

Tables 6, 7 summarize the comparison among the eight scenarios in terms of the mean perceived autonomy and reactance and the application-specific effects of awareness and explanation (also see Figures 4, 5 for visualization of the results). These results revealed similar differences across the application scenarios in terms how participants perceived autonomy and experienced reactance in those scenarios. We highlight a few noteworthy results below.

**Table 6:** The means and standard deviations of perceived autonomy and the modeling results (regression coefficients with standard errors and adjusted *R*^2^) for each of the eight application scenarios (ranked from high to low in terms of perceived autonomy).

	**Mean (*SD*)**	**Effect of awareness**	**Effect of explanation**	**Interaction effect**	**Adjusted *R^**2**^***
Fitness coach	3.88 (0.65)	0.08 (0.04)	−0.07 (0.04)	0.04 (0.04)	0.023
Car navigation	3.81 (0.77)	−0.02 (0.04)	**0.26 (0.04)**	−0.03 (0.04)	0.107
E-mail correction	3.68 (0.79)	−0.06 (0.04)	0.04 (0.04)	−0.01 (0.04)	−0.0001
Travel booking assist	3.53 (0.85)	0.10 (0.05)	0.04 (0.05)	0.08 (0.05)	0.017
Movie recommender	3.52 (0.81)	−0.08 (0.04)	0.12 (0.04)	−0.01 (0.04)	0.022
Online shopping	3.51 (0.76)	−0.002 (0.04)	0.08 (0.04)	0.04 (0.04)	0.006
Climate control	3.48 (0.83)	−0.003 (0.05)	−0.10 (0.05)	0.05 (0.05)	0.010
Social media	3.32 (0.82)	−0.01 (0.05)	−0.03 (0.05)	0.12 (0.05)	0.013

*Significant effects are highlighted in bold font (p < 0.00625)*.

**Table 7:** The means and standard deviations of reactance and the modeling results (regression coefficients with standard errors and adjusted *R*^2^) for each of the eight application scenarios (ranked from low to high in terms of reactance).

	**Mean (*SD*)**	**Effect of awareness**	**Effect of explanation**	**Interaction effect**	**Adjusted *R^**2**^***
Fitness coach	2.57 (0.80)	−0.05 (0.04)	0.06 (0.04)	0.01 (0.04)	0.001
Car navigation	2.58 (0.84)	−0.03 (0.05)	–**0.18 (0.05)**	−0.005 (0.05)	0.038
Climate control	2.71 (0.83)	0.08 (0.04)	**0.13 (0.04)**	−0.08 (0.04)	0.032
Movie recommender	2.89 (0.86)	**0.14 (0.05)**	0.01 (0.05)	−0.05 (0.05)	0.020
Travel booking assist	2.92 (0.82)	−0.06 (0.05)	0.01 (0.05)	−0.09 (0.05)	0.010
E-mail correction	2.92 (0.83)	0.06 (0.05)	−0.04 (0.05)	−0.02 (0.05)	−0.0003
Online shopping	3.03 (0.82)	0.09 (0.04)	0.03 (0.04)	−0.04 (0.04)	0.006
Social media	3.11 (0.76)	0.08 (0.04)	0.07 (0.04)	−0.09 (0.04)	0.024

*Significant effects are highlighted in bold font (p < 0.00625)*.

First, regardless of the specific design features of awareness of the presence of intelligence and providing explanation, some applications tended to be perceived as less autonomy-threatening and/or to induce less psychological reactance. More specifically, participants seemed to perceive higher autonomy when interacting with the car navigation system and the fitness recommendation system. These two AI applications also induced less psychological reactance than other applications. In contrast, the application that filtered content in social media was perceived to be more autonomy-threatening and it induced higher level of reactance among our participants. The perceived autonomy and reactance for the remaining applications were at levels ranging between these three more extreme cases.

Second, the application scenario of the car navigation system also stood out for the effects of explanation on autonomy and reactance. The effect sizes, in terms of both the regression coefficients and the percentage of variance explained (Adjusted *R*^2^) were substantially larger than the estimates for all other applications. In particular, the amount of variance explained mainly by the explanation manipulation was about 10% of the total variance in perceived autonomy, a number that was at least five times higher than the corresponding figures for other applications. To interpret the effect size from a different perspective, providing explanation when suggesting driving routes could increase perceived autonomy by around half a point and decrease reactance by about 0.36 on five-point scales.

Third, psychological reactance was significantly influenced by either awareness or explanation in two application scenarios. For the movie recommender, the results suggested that making users aware of AI behind the system might not be a good idea as it resulted in higher level of reactance. For the automatic house climate control system, providing an explanation seemed to backfire as it actually induced more psychological reactance from the participants.

## Discussion

### General Perception of Autonomy and Reactance

**Figure 6:**
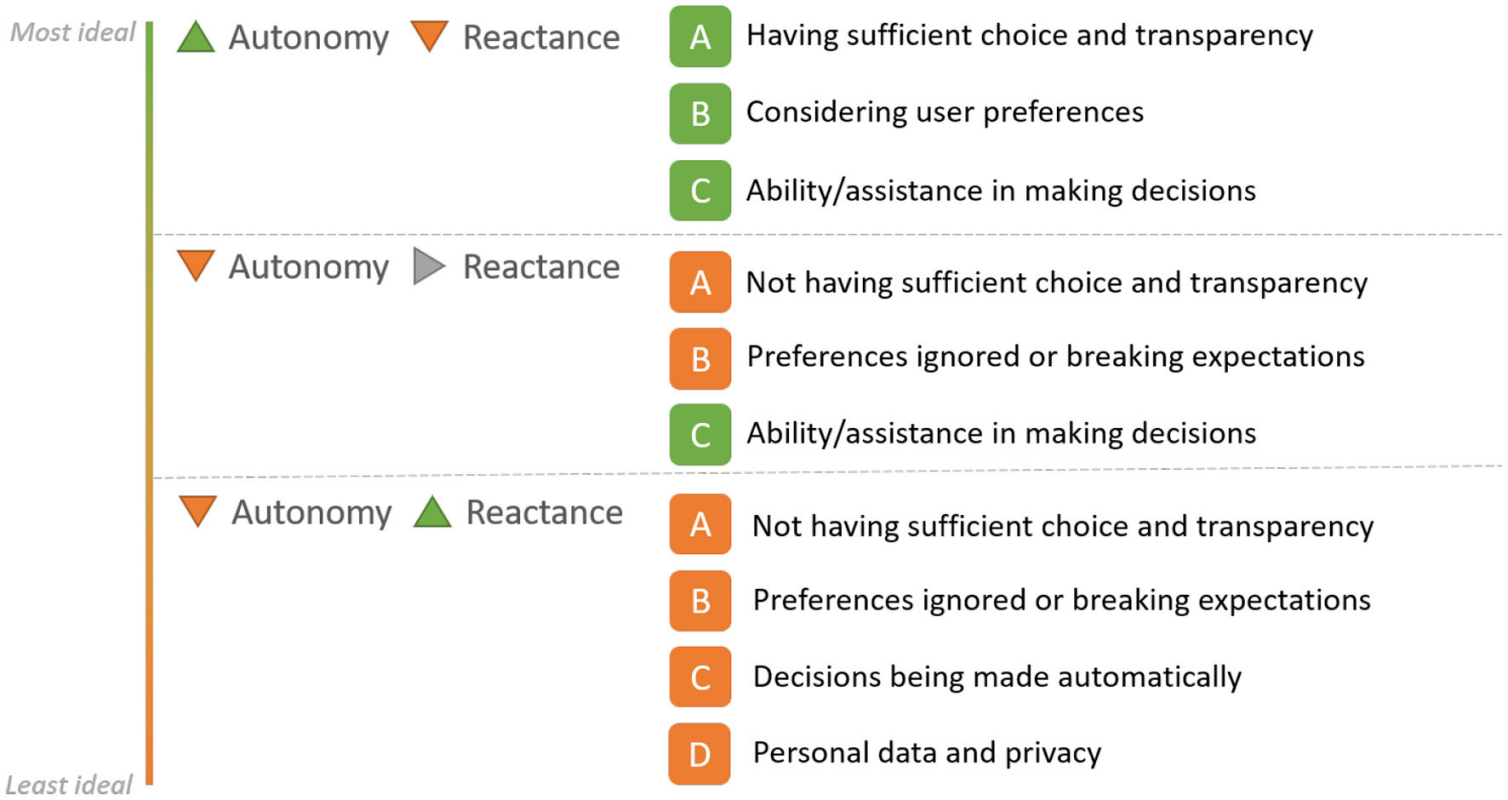
Summary of inferences drawn from the study on different factors that influence the perception of autonomy and reactance.

Based on the outcomes of our study, we identified that four main factors primarily contributed to perceptions of autonomy or reactance which are in line with similar or related factors identified by researchers in other studies (Eiband et al., 2019; Sankaran and Markopoulos, 2021). These factors are (A) having sufficient choice and transparency, (B) considering user preferences, (C) ability or assistance in making decisions, and (D) personal data and privacy. While the extent of influence of these factors on autonomy perception varied across different application contexts, a few general inferences could be drawn. Overall, when factors A, B, and C were present, participants perceived higher autonomy and lower reactance which is most ideal when interacting with AI applications (Figure 6). On the other hand, when factors A and B were not present, autonomy was correspondingly reduced. However, with factor C being present, it did not necessarily have an impact on reactance which remained neutral. Finally, not having any of the factors, compounded with the lack of understanding of how personal data and privacy were managed (D), there was a considerably lower perception of autonomy with increased psychological reactance.

We will discuss these variations in autonomy and reactance perceptions by reflecting upon user perceptions across different application contexts as gleaned from participants' responses to the open-ended questions at the end of the survey.

#### Higher Autonomy and Lower Reactance

In most application contexts where participants perceived that users' preferences were taken into consideration and had sufficient choices, they rated a higher perceived autonomy. In the context of navigation or thermostat, where users found it difficult to make an independent decision, the factor on *assistance* to the decision making still contributed to a higher degree of perceived autonomy. These perceptions were irrespective of whether they were made aware of the presence of AI or received explanations.

“*Computing routes is a task best done by algorithms rather than* *human experience**.” [P283; not made aware of AI presence, received explanation]*

“*I liked the health app the most as I felt like it made the choice of what to recommend* *on the basis of the user's goal**, but still seemed to provide an opportunity to scroll further down and explore other options.” [P74; made aware of AI presence, did not receive explanation]*

“*The application that adjust the thermostat of the house, because it's something that* *most people don't know how to do it properly* *with their own logic.” [P107; not made aware of AI presence, did not receive explanation]*

#### Lower Autonomy but no Reactance

People perceived a lower sense of autonomy in the thermostat scenario, but they did not report any reactance. The lower autonomy perception could indicate that people feel their personal preferences are not considered but do not necessarily show reactance because they are unsure of the ideal decision. These are reflected in the following opinions.

“*Thermostat one. It took a lot of factors into consideration when deciding the perfect room temperature*. *It didn't however let the user apply their personal preferences**.” [P88; not made aware of AI presence, received explanations]*

#### Lower Autonomy and Higher Reactance

Finally, we observed that people perceived a lower degree of autonomy in the social media context and exhibited greater reactance. The users cited various reasons for this perception: invasiveness, lack of understanding of data usage, and streamlining or restricting them to limited content.

“*The twister application (the fictitious social media interface) … we are not sure if this site will not* *use our data for some purposes*”* [P96; made aware of AI presence, received explanations]*

“*the social media one, since* *it restricts the owner to certain topics**, therefore they are unable to look up new topics.” [P122; made aware of AI presence, did not receive explanations]*

“*The social media feed because* *it was too invasive* *in the way it altered the feed.” [P123; made aware of AI presence, received explanations]*.

These perspectives are in line with certain user perceptions revealed in the study of algorithmic curation of Facebook's news feeds where users felt that the algorithm made decisions on which post to show them or violated their expectations (Rader and Gray, 2015).

### Effect of the Presence of AI and Explanations on Autonomy Perception and Reactance

As AI-based applications are getting increasingly pervasive and embedded in our daily lives, an increasing need and emphasis are being laid on designing AI systems that are transparent, accountable, and interpretable (Barredo Arrieta et al., 2020). The need for explanation has also been cited as necessary to support trust and acceptance of decisions made by AI applications (Ribeiro et al., 2016; Alexandrov, 2017). In everyday interactions, specifically in contexts where people seek to understand decisions or local predictions made by the system, they seek to understand “why” the predictions or decisions were made (Liao et al., 2020; Sankaran and Markopoulos, 2021). Algorithmic anxiety and aversion have also been cited as growing concerns of interacting with AI in everyday contexts (Dietvorst et al., 2015; Jhaver et al., 2018).

Based on these premises we expected to find a substantial difference in people's perception of autonomy and a greater reactance when they were made aware of interacting with an intelligent algorithm or when it was not transparent to them as to why specific recommendations were made. Some participant opinions reflected this expectation (e.g., P112).

“*the social and the one to buy things on the internet [referring to social media and online shopping applications], I say for both I did not like them because I like to choose for myself what to buy or the latest news” [P112; made aware of AI presence, did not receive explanations]*

Nonetheless, we did not find an overall significant effect of awareness or explanations on perceptions of autonomy and reactance except in the car navigation and the travel booking contexts. We believe that there could be a couple of factors that caused this outcome which is detailed in the following sub-sections.

#### Real-Time Action and Consequences

We explored diverse AI-based applications, and each may impact our everyday lives and activities differently. For example, the recommendations of the navigation system or thermostat are received in real-time and the effect is perceived almost instantly, and it directly influences our subsequent actions.

“*I think the car navigation app. First it provided familiar routes based on their navigating history, which I find very smart! Then on real time made suggestions on changes of routes based on real life inconvenients [sic], like a closed street for example. I think that this app was the most helpful.” [P100, made aware of AI presence, received explanations]*

On the other hand, the movie recommender application does not directly or immediately impact our actions or pose any consequences should we not adhere to the recommendations. Therefore, the need to understand *how* and *why* the recommendations are made, might be more important in supporting autonomy when the consequences are experienced in real-time and where an action based on the recommendation is expected. It would be useful for future studies to examine more systematically the impact of explanations when real-time actions are involved.

#### Human-Likeness

In recent studies, human-likeness has been shown to increase trust when interacting with intelligent agents and reduce reactance (Kulms and Kopp, 2019; Pizzi et al., 2021). The travel booking assistant was presented as a conversational chatbot rather than a recommender system in our study. The conversational nature of the booking assistant could have been perceived as interacting with a human rather than an algorithm (as evident from the quote of P35, who was not made aware of AI presence). Furthermore, participants could have experienced empathy wherein they felt that the system considered their preferences (in the case of both P62, who was primed, and P35, who was not primed).

“*I think the most intelligent app was something like booking.com, great predictions about our next trip and place to stay, taken on the basis of the past.” [P62; made aware of AI presence, received explanations]*

“*The assistant to book a hotel because she knew his preferences, she was easy to use and she booked the hotel very quickly.” [P35; not made aware of AI presence, received explanations]*

While we did not systematically vary the human-likeness in all different contexts, it might be a valuable follow-up of this study to assess perceptions of autonomy in future studies.

### Implications for Future Research

The overall reflections discussed in section ‘General Perception of Autonomy and Reactance’ informs us that it is important to consider the four key factors when designing AI-based applications and interactions in everyday contexts to enhance people's perceptions of autonomy and minimize reactance: providing sufficient choice and transparency, considering user preferences, providing users with the ability or assistance to make independent decisions and respecting personal data privacy. To achieve this while reaping the benefits and advantages that AI offers, future research could investigate how to find an optimal balance between human autonomy and machine autonomy through approaches such as a human-in-the-loop or machine-in-the-loop approaches, symbiotic AI, hybrid intelligence, and other similar collaborative approaches (Kamar, 2016; Veloso, 2018; Inkpen, 2020).

Likewise, future research will have to carefully consider how AI awareness and explainability is built into the design of AI and interactions. A more systematic approach might have to be adopted to identify how human-like or naturalness in interactions could impact perceptions of autonomy or reactance. Moreover, when aiming for transparency, it is important to consider how variables of explanability such as why, why not or how can influence these perceptions as well. Some potential trends and trajectories in that direction has been identified by Abdul et al. (2018).

### Limitations and Future Work

This survey study is a first step in exploring how human autonomy is impacted in everyday lives with growing interactions with AI-based applications.

The reason for adopting a video-based design fiction method was primarily to foreground people's experience of an interaction rather than gather insights over function and usability as adopted in prior research (Briggs et al., 2012). This approach enabled us to gather interesting insights on people's perception of autonomy and reactance across various potential application contexts and scenarios, although the differences were not largely significant. A future study including more active user interaction from across a longer duration might reveal more significant differences and shed light on *experienced* autonomy and reactance as opposed to *perceived* autonomy and reactance. Furthermore, the manipulation of making users aware of the presence of AI could have been stronger. This could have potentially been a reason why we did not see significantly higher reactance in many contexts as suggested in literature.

Computer scientists are focusing on developing algorithmic transparency and glass-box machine learning models (Abdul et al., 2018; Hayes and Moniz, 2021). On the other hand, researchers have been proposing design guidelines and considerations to build explainable interfaces and increase AI literacy among users (Amershi et al., 2019; Liao et al., 2020). Based on the outcomes of our exploratory study, we believe that future research must consider the application context and the nature of interaction before delving into designing explanations or increasing awareness of AI among users.

Future research can also study in more depth the impact on autonomy in specific application contexts and explore how perceptions vary when users have a more active interaction with the applications. By opening our data and highlighting user perceptions across diverse contexts, we believe that researchers can build upon these insights to develop AI-based applications that respect human autonomy and reduce psychological reactance.

## Conclusion

We presented an exploratory study to understand user perceptions of autonomy and reactance in different everyday application contexts of AI. We found that in contexts that support real-time decision making, giving multiple choices to users, and considering their preferences, participants reported a greater sense of autonomy and lower reactance. Conversely, in contexts where choices were restricted or streamlined, and the expectations of participants were violated, people reported greater reactance and lower autonomy. Furthermore, we observed that common approaches of algorithmic transparency through explanations or increasing user awareness of AI did not evenly influence their perception of autonomy or reactance across different contexts. Future research could draw on these insights and consider the application context and the level of user participation when developing AI applications to respect human autonomy.

## Data Availability Statement

The raw data from the study, power and data analysis scripts as well as the design fiction for the different application contexts and experimental conditions can be found online on the Open Science Framework repository: https://osf.io/er6zy/?view_only=bc1d6f6622d14798aee2b52e6725d650.

## Ethics Statement

The studies involving human participants were reviewed and approved by Ethical Review Board Department of Industrial Design Eindhoven University Of Technology, Netherlands. The patients/participants provided their written informed consent to participate in this study.

## Author Contributions

The overall research question was framed by all authors. SS contributed in creating the design fictions and carried out the experiment. CZ performed the statistical analysis. HA and PM mentored the process and contributed to the critical reflections through the process. All authors contributed to the manuscript.

## Funding

The study and work described in this manuscript were funded by the Human-AI Alliance between Eindhoven University of Technology, Utrecht University, and University Medical Center of Utrecht, Netherlands. https://human-ai.nl.

## Conflict of Interest

The authors declare that the research was conducted in the absence of any commercial or financial relationships that could be construed as a potential conflict of interest.

## Publisher's Note

All claims expressed in this article are solely those of the authors and do not necessarily represent those of their affiliated organizations, or those of the publisher, the editors and the reviewers. Any product that may be evaluated in this article, or claim that may be made by its manufacturer, is not guaranteed or endorsed by the publisher.

## Footnotes

^1^ https://www.prolific.co/
